# Transpiration Response of Cotton to Vapor Pressure Deficit and Its Relationship With Stomatal Traits

**DOI:** 10.3389/fpls.2018.01572

**Published:** 2018-10-30

**Authors:** Mura Jyostna Devi, Vangimalla R. Reddy

**Affiliations:** ^1^Adaptive Cropping Systems Laboratory, Beltsville Agricultural Research Center, United States Department of Agriculture-Agricultural Research Service-Northeast Area, Beltsville, MD, United States; ^2^Oak Ridge Institute for Science and Education, Oak Ridge, TN, United States

**Keywords:** cotton, gas exchange, stomata, transpiration, vapor pressure deficit, water potential

## Abstract

Many studies have demonstrated that the cotton in warm environments is vulnerable to water-limitations thus reducing the yield. A number of plant traits have been recommended to ameliorate the effects of water deficits on plant growth and yield. Limitation on maximum transpiration rate (TR) under high vapor pressure deficit (VPD), usually occurs during midday, is often considered as a water conservation trait. The genotypes with this trait are desirable in high VPD environments where water deficits commonly develop in the later part of the growing season. Our objective of the study was to find the genotypic variation for the trait limited TR under high VPD and also to study leaf temperature, water potential, photosynthesis, and stomatal conductance responses. Also, our objective was also to study the structural changes in the stomatal traits when exposed to long term high VPD conditions and involvement in such responses. In the present study, 17 cotton genotypes were studied for their (TR) response to various VPD environments under well irrigated conditions. Out of 17, eight genotypes limited TR after approximately 2 kPa VPD and rest of them increased their TR with increased VPD. Five selected genotypes with different TR response to increasing VPD were further studied for gas exchange and stomatal properties. All genotypes, irrespective of exhibiting limited TR at high VPD, reduced stomatal conductance, photosynthesis and water potential at high VPD of 3.3 kPa. The genotypes with limited TR modified their stomatal traits mostly on the adaxial surface with frequent and small stomata under high VPD. The genotypes with limited TR also exhibited an increase in epidermal cell expansion and stomatal index at contrasting VPD gradients to effectively balance the liquid and vapor phase conductance to limit TR at high VPD.

## Introduction

Drought is the major limiting factor for cotton production, affecting growth, productivity, and fiber quality (Parida et al., 2007). Nearly one-third of the arable land around the globe suffers from water limitation, which is estimated to double by 2050 (Vicente-Serrano et al., 2012). Therefore, it is essential to produce or identify drought tolerant varieties to improve cotton yield in water-limited as well as well-watered environments. Nevertheless, only a few plant traits have actually been considered to improve drought resistance despite the many physiological traits involved in plant responses to drought. Several adaptive strategies have evolved in plants to cope with drought stress especially in environments where water deficit occurs frequently. One of the strategies is to limit transpiration rate (TR) under high vapor pressure deficit (VPD) environments. VPD, is the difference between the amount of moisture in the air and the moisture air can hold when it is saturated. It combines the effect of temperature and RH, has an effect on transpiration of the plants. High VPD, which usually occurs usually in the midday to end of the day, influences the water balance of the plant thus affecting photosynthesis and growth. Limiting transpiration in this situation will help the plant to conserve water for use later in the crop growing seasons when drought develops. Limited TR in response to high VPD has been observed in several crop species due to genotypic variability (Fletcher et al., 2007; Devi et al., 2010; Gholipoor et al., 2010; Kholová et al., 2010; Schoppach and Sadok, 2013) and found to increase crop yield by 75% in water-limited areas based on simulations in crop models (Sinclair et al., 2005, 2010; Sermons et al., 2012; Drake et al., 2013; Shekoofa et al., 2016).

High VPD under water limited conditions worsen the stress effects on plants either by increasing the TR (anisohydric) or by reducing carbon uptake (isohydric). Even though high VPD in general increases the diffusion process, plants regulate transpiration through stomatal closure or by means of other physiological responses (Aasamaa and Sõber, 2011). Stomata regulate water status of the plant conservatively by regulating the transpiration so that it matches to the atmosphere and the proportions of the soil–plant hydraulic system (Attia et al., 2015). Plants reduce stomatal conductance in response to high VPD between the leaf and atmosphere to maintain water potential as a feedforward mechanism (Bunce, 1997). By reducing stomatal conductance to water vapor, plants minimize water loss and maintain hydration of plant cells as VPD increases. There are many studies showing that the high VPD reduces stomatal conductance thereby affecting photosynthesis and growth (Wong, 1993; Bunce, 1997; Ottosen et al., 2002; Bunce, 2003; Ben-Asher et al., 2013). On the contrary, some studies have proposed that this high VPD impact on stomatal conductance may not influence photosynthesis (Carins Murphy et al., 2014; Shibuya et al., 2017).

Irrespective of the fact that the conductance of water flux depends on the atmospheric conditions surrounding the leaf, it is the leaf anatomy that controls the conductance of liquid water and water vapor (Carins Murphy et al., 2014). Stomata are gateway for gas exchange between the leaf and the surrounding environment. The equilibrium of water use and carbon assimilation in leaves basically relies upon water transport through the plant and the resultant coordination with the stomatal system (Kaiser, 2009; Brodribb and Jordan, 2011). The differences in stomatal anatomy and structure influence transpiration and photosynthetic activities demonstrating adaptations of the plants to various environmental variables (Larcher, 2003). Many studies have shown the modification of stomatal density or anatomical changes in stomata in response to various environmental factors such as CO_2_ (Driscoll et al., 2006), drought (Galmés et al., 2007; Xu and Zhou, 2008), light (Martins et al., 2014) and evaporative demand (Aliniaeifard and van Meeteren, 2014). Plasticity of stomatal, epidermal and vein density in response to variation in VPD has been observed in some studies (Torre et al., 2003; Carins Murphy et al., 2014). Irrespective of several studies on stomatal physiology, the complex mechanisms involved in the adjustment of stomatal traits such as size and aperture which regulate stomatal conductance are still poorly understood. In cotton, VPD is considered as a major promoting factor for TR (Isoda and Wang, 2002). Differences in TRs of cotton were observed in humid and arid conditions with increasing transpiration in arid environments (Isoda and Wang, 2002; Wang et al., 2004; Duursma et al., 2013; Conaty et al., 2014). Both TR and stomatal conductance showed strong response to varying VPD and photosynthesis was relatively insensitive (Duursma et al., 2013). However, no studies have investigated cotton genotype differences in TR response to various VPD environments. Hence, our objective in this study was to identify the genotypes limiting TR under high VPD and identify genotypic variation for this trait in cotton, which can be implemented in the development of drought tolerant varieties. Also, to understand the physiological responses involved to control TR, especially stomatal characters. In this study, therefore, we report observations on the response of transpiration of 17 cotton genotypes to differing VPD levels. Five genotypes with differences in their transpiration response to different VPD levels were selected out of 17 for a detailed study. The selected genotypes with differential VPD-TR response were subjected to high and low VPD levels to determine differences in the response patterns of the stomatal system. Our hypothesis is that the genotypes with differences in their TR response to VPD will show differences in their stomatal properties. The cultivars that limit TR at high VPD may modify stomatal and epidermal features when acclimated to high VPD in order to manage their TR. Conversely, the same difference would not be prominent in genotypes whose TR response to increasing VPD was linear and not restricted at higher VPD.

## Materials and Methods

Total of three experiments was conducted to study the TR response to VPD and stomatal traits of cotton. Out of the three experiments, two experiments were to study the TR response of 17 cotton genotypes to VPD. The third experiment was conducted to study the stomatal traits of the selected genotype, after acclimatization of the genotypes for 10 days to low and high VPD environments. Gas exchange and water potential parameters were also observed in the same experiment. The experimental details from 1 to 3 were detailed in Table 1.

**Table 1:** Average temperature (°C) and RH (%) obtained in growth chambers and the average VPD calculated from temperature and RH data in all three experiments.

Temperature (°C)	RH (%)	VPD (kPa)	Number of genotypes	Parameters measured
23 ± 0.4	74 ± 2	0.74 ± 0.01	17 genotypes	Transpiration response to different VPD levels (plants were grown in the greenhouse for 30 days, and experiments were conducted in growth chambers)
24 ± 0.2	71 ± 3	0.86 ± 0.02		
25 ± 0.1	68 ± 5	1.00 ± 0.01		
25 ± 0.5	67 ± 4	1.03 ± 0.01		
25 ± 0.1	64 ± 4	1.15 ± 0.01		
26 ± 0.3	55 ± 3	1.50 ± 0.02		
27 ± 0.2	51 ± 3	1.75 ± 0.03		
27 ± 0.5	48 ± 3	1.87 ± 0.01		
28 ± 0.5	48 ± 3	1.92 ± 0.01		
28 ± 0.5	45 ± 3	2.08 ± 0.02		
29 ± 0.4	42 ± 5	2.33 ± 0.02		
30 ± 0.3	39 ± 5	2.57 ± 0.02		
32 ± 0.3	35 ± 2	3.02 ± 0.03		
**Experiment 3**				
24 ± 0.3	70 ± 2	0.83 ± 0.01	5 genotypes	Water potential, leaf temperature, gas exchange parameters, stomatal and epidermal cell structure (plants were grown in the greenhouse for 23 days, and experiments were conducted in growth chambers)
26 ± 0.4	60 ± 2	1.33 ± 0.01		
27 ± 0.2	55 ± 5	1.66 ± 0.02		
28 ± 0.4	50 ± 4	1.87 ± 0.01		
31 ± 0.3	40 ± 5	2.65 ± 0.01		
32 ± 0.5	30 ± 5	3.26 ± 0.02		

*Number of genotypes and parameters measured in each experiments in three experiments.*

### Screening of Genotypes for TR Response to VPD (Experiments 1 and 2)

Seventeen cotton genotypes (Table 1) were selected for this investigation and seeds of these genotypes were obtained from USDA-ARS, Lubbock, Texas. Before the measurement of VPD response, the plants were grown in pots in a greenhouse at the Beltsville Agriculture Research Center (BARC), ARS-USDA, and Beltsville, MD, United States. The pots (21 cm diameter and depth) were filled with Vermiculite (Miracle-Gro Lawn Products, Inc., Marysville, OH, United States) up to brim and planted with two seeds per pot. After about a week, each pot was thinned to one plant. The plants were grown under well-watered conditions, and the air temperature in the greenhouse was regulated at 27°C/21°C (day/night). Humidity in the greenhouse was not controlled, and the values measured were in between 60 and 70% in all three experiments during the daytime. Humidity in the greenhouse during night time was between 70 and 80%. The calculated VPD values in the greenhouse ranged from 1.00 to 1.45 kPa during daytime and in the night around 0.6 kPa. Grow lights were used from 6 to 9 AM as a supplement to natural day light received by the plants to bring the total light received by the plants to 1000 μmol m^-2^ s^-1^. Plants were also watered with full strength Hoagland nutrient solution every alternate day. Approximately after 30 days (6–7 true leaf stage), the plants were moved to growth chambers where different temperatures and humidity levels were set to maintain desired VPD levels as listed in Supplementary Table S1. The photosynthetic photon flux density (PPFD, 16 h d^-1^) of 800 μmol m^-2^ s^-1^ was maintained in each chamber. The humidity levels are maintained by using different air sources. To maintain low humidity powerex 40 hp oil-less rotary scroll air compressors (Powerex, Harrison, OH, United States) were used to dry the air. For the high humidity full cone misting nozzles (McMaster-Carr, Princeton, NJ, United States) were used and these nozzles provided small atomized spray drop lets for the humidification of the chamber to create desired high humidity levels. The maintained temperatures and humidity levels in the growth chambers in each experiment and the corresponding VPDs were listed in Supplementary Table S1. The plants were acclimated to growth chamber 1 day before transpiration measurements began. In all the growth chambers the temperature was set to the 27°C/21°C (day/night) as maintained in the greenhouse. The transpiration response was measured gravimetrically in all 17 genotypes in experiments 1 and 2 with six replicates in each experiment. In the evening of the day before the measurements began all plants were watered to saturation to avoid any water limitation stress.

In the morning of the day of measurements, the soil surface was covered with aluminum foil to avoid soil evaporation during the measurements of plant water loss. To avoid the effect of the growth chamber the plants were shuffled between the growth chambers before the measurements. The plants subjected to acclimation for an hour to each VPD and then the initial weight of the plant was recorded by weighing. The plants were exposed for an hour to each VPD level and then reweighed to measure the final weight. The difference between initial and final was calculated as transpiration of the plant. The same procedure was continued for all VPD levels and the plants were harvested. The leaf area of each plant was measured using leaf area meter (LI- 3100, Li-Cor, Lincoln, NE, United States). The TR of each plant was calculated as a ratio of transpiration per leaf area. Due to the limited space available in the growth chamber, 17 cotton genotypes were divided into three sets. First and second set with six genotypes in each set and third set including the rest of the five genotypes. The experiments were run with six replicates per genotype. In each set, a total of six chambers were used, each chamber including one replicate from each genotype.

### Investigation of Stomatal Traits in Response to VPD (Experiment 3)

#### Leaf Gas Exchange

Leaf gas exchange parameters were measured only for the selected genotypes in the third experiment. A total of five genotypes were selected from experiments 1 and 2 based on their TR response to VPD. In the third experiment, the plants were grown in the greenhouse as mentioned above in experiments 1 and 2 until 23 days after emergence (vegetative stages 4–5 true leaf stage) and then moved to growth chambers where the experiment was conducted. Six VPD treatments 0.9, 1.3, 1.7, 1.9, 2.7, and 3.3 kPa were maintained in six chambers and a total of three replicates per genotype per treatment was included. The temperatures and humidity levels maintained in each chamber were listed in Supplementary Table S1. The photosynthetic photon flux density (PPFD, 16 h d^-1^) of 800 μmol m^-2^ s^-1^ was maintained in each chamber. The measurements were taken after 10 days of transferring the plants to the growth chamber. The plants were not rotated among the chambers as constant VPD was maintained in each chamber where there might be an effect of the growth chamber.

The gas exchange parameters were measured on a fully developed youngest leaf (7–8 true leaf stage), which had developed (after unfolding) after transferring the plants to growth chamber of particular VPD in each genotype. Net photosynthetic rate per unit leaf area (*A*), stomatal conductance (*g*_s_), and TR (*E*) were measured using a 0.26 chamber connected to a portable photosynthesis system (LI-6400, Li-Cor, Inc., Lincoln, NE, United States). The temperature of Li-Cor leaf chamber were closely matched to the growth chamber and CO_2_ level was set at 400 ppm. The flow rate of Li-Cor was adjusted between 300 and 500 μmol s^-1^ to obtain the close RH levels as in growth chamber (see Supplementary Table S1).

### Leaf Water Potential (Ψ) and Leaf to Air Temperature Differences (LT-T°C)

Leaf water potential was measured at midday (11.30–12.30 h) using the youngest, fully expanded leaves (those for which gas exchange was measured) to different VPDs after taking the gas exchange and leaf temperature measurements. A Wescor psypro meter (Wescor, Pullman, WA, United States), eight channel water potential data logger was used for leaf water potential measurement, using three replicates for each treatment per genotype. The leaf temperature was estimated by using handy Infrared meter Flir TG165 (Flir systems, Inc., Nashua, NH, United States) by pointing to the whole leaf. The difference between leaves to air temperature was calculated as the difference between leaf temperature measured and the air temperature maintained in the growth chamber.

### Stomatal Traits

Stomatal traits were measured in the five genotypes in the plants exposed to low VPD of 0.9 kPa and high VPD of 3.3 kPa. The impressions were collected from the leaves selected for which gas exchange was measured. The impressions for stomatal traits were taken after measuring gas exchange, leaf temperature and leaf water potential. The impression approach was used to determine leaf stomatal properties. The adaxial and abaxial epidermis of the leaf was cleaned using a degreased cotton ball and then carefully layered with nail varnish in the mid-area between the central vein and the leaf edge, for approximately 1–2 min (Xu and Zhou, 2008). The thin film (approximately 10 mm × 10 mm) was peeled off from the leaf surface using clear tape and then immediately stick on to the microscopic slide. Numbers of stomata and epidermal cells for each film strip were counted using Nikon Eclipse E600 (Nikon Inc., Melville, NY, United States) equipped with a Nikon Digital Camera DXM1200. The area of the stomata and the epidermal cell was measured using Nikon NIS elements D. The length and width of the stomatal aperture were also measured using the analytical parameters of the NIS elements D. The stomatal density (SD) and epidermal cell density (ED) were expressed as the number of stomata per unit leaf area. The stomatal ratio was calculated as the ratio of stomata from adaxial leaf surface to the abaxial leaf surface. The leaf stomatal index was estimated using the formula [*s*/(*e* + *s*)] × 100, where s is stomatal density and e is epidermal cell density (Xu and Zhou, 2008).

### Statistical Analysis

Transpiration data of each genotype from the two experiments exposed to each VPD level were averaged (*n* = 12) and plotted against corresponding VPDs obtained. A two-segment linear regression (Prism 6.0, GraphPad Software Inc., San Diego, CA, United States) for TR vs. VPD was performed for all the genotypes. If the slopes were not significantly different (*p* < 0.05) using segmental linear regression, a simple linear regression was applied to all the data. The genotypes expressing the two-segment response, the regression analysis yielded the VPD breakpoint (*X*_0_) between the two linear segments as well as the slope of each segment. The standard error for the VPD breakpoint and the two slopes was an output of the regression analysis. The gaseous traits (*n* = 3) measured at different VPDs were also averaged for each genotype to perform two-segmental linear regression. If the regression was not obtained then a simple linear regression was used.

Water potential, LT-T and all stomatal traits were analyzed using ANOVA and the difference between the means of the genotypes for all the parameters was performed using Tukey’s Kramer test.

## Results

### Genotypic Variation in TR Response to VPD

Seventeen genotypes of cotton were screened for their TR response to VPD in two repeated sets of experiments. In both the experiments, the distribution of temperature and relative humidity during the experiments was in the range of 23–32°C and 35–75%, respectively. The range of VPD treatments including all genotypes was between 0.74 ± 0.01 and 3.02 ± 0.03 kPa. The variation in VPD was mainly due to variation in the humidity levels established in the chambers as a result of differing air sources (see section “Materials and Methods”). The desired levels of VPD were obtained in each experiment (Table 1).

**FIGURE 1:**
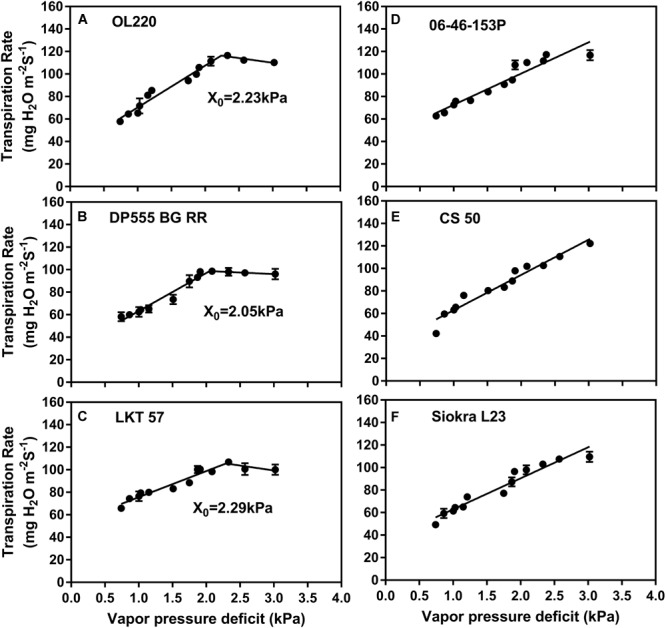
Transpiration rate (TR) of cotton genotype to vapor pressure deficit (VPD). TR (mg H_2_O m^-2^ s^-1^) response to increasing VPD (kPa) of the six cotton genotypes. **(A)** OL220 **(B)** DP555 BG RR and **(C)** LKT 57 represents genotypes with two segmental regression. The genotypes increased TR until VPD breakpoint (BP) *X*_0_ (kPa) and then reduced TR. The values of *X*_0_ indicated in each panel from **(A–C)**. Panels from **(D–F)** represents genotypes 06-46-153P, CS 50 and Siokra L23, respectively, with linear TR response to increasing VPD.

A clear distinction among genotypes in the response of TR to VPD was observed (Table 2). Some genotypes were well characterized by the two-segmental analysis with a break point (BP) (*X*_0_), and other genotypes exhibited a linear increase in TR for the range of VPD tested (Figure 1 and Table 2). Out of the 17 genotypes tested, eight were found to display a BP in TR increase as VPD was increased (Table 2). An example of two segmental linear regression was provided in the Figures 1A–C. The *R*^2^ for all data of each of these eight genotypes based on the two-segment regression ranged from 0.86 to 0.98. The value for the BP ± SE ranged from 1.58 ± 0.24 to 2.29 ± 0.15 kPa, with an average of 1.98 ± 0.09 kPa for the eight genotypes. The secondary slope of these eight genotypes above the BP ranged from -11.7 to + 14.7 mg H_2_O m^-2^ s^-1^ kPa^-1^. The distribution of slopes among eight genotypes were six positive and two negatives implying limited TR after BP (Table 2).

**Table 2:** Outputs from analysis of the two-segment linear regression and linear regression models for 17 cotton genotypes tested in experiments 1 and 2.

Genotype	Limiting transpiration	Slope 1 (mg H_2_O m^-2^S^-1^)	*X*_0_ (kPa)	Slope 2 (mg H_2_O m^-2^S^-1^)	Intercept	*R*^2^
**05MMH**	No	24.66	–	–	46.42	0.867
**06-46-153P**	No	27.78	–		44.80	0.915
**12-8-103S**	**Yes**	34.38	2.21	–11.71	21.63	0.958
**CS 50**	No	31.38	–	–	31.49	0.946
**DeRiddler (Red-leaf)**	No	34.23	–	–	27.32	0.937
**DP555 BG RR**	**Yes**	33.78	2.051	–2.086	29.44	0.973
**FiberMax 2870GLB2**	No	25.58	–	–	33.18	0.970
**FiberMax 9180**	**Yes**	39.61	1.578	0.8946	20.63	0.923
***G*. *arboreum***	No	34.05	–	–	25.09	0.984
**LKT 57**	**Yes**	23.18	2.292	–8.774	52.4	0.942
**MC 220**	**Yes**	39.43	1.79	–2.187	27.4	0.941
**OL220**	**Yes**	37.24	2.23	–8.42	32.99	0.971
**PHY 72**	**Yes**	27.75	1.841	–2.021	37.8	0.930
**Siokra L23**	No	27.57	–	–	35.2	0.938
**ST 5599BR**	No	36.11	–	–	21.1	0.985
**TM-1**	No	34.37	–	–	38.3	0.970
**TX-1151**	**Yes**	27.68	1.87	14.72	35.2	0.941

*Eight genotypes fitted the two segmental linear regression and are their slope 1, break point (X_0_) and slope 2 were obtained. Nine genotypes fitted the single linear model and their only single slope value was listed. The genotypes in the table were listed alphabetically.*

The VPDvsTR data of nine genotypes did not fit the two-segment model, and hence, a single linear regression model was used (Figures 1D–F and Table 2). The linear regression generally fit these data well with the *R*^2^ ranging from 0.76 to 0.92. The genotypes transpired with an increase in VPD in the range of 24.6 to 36.1 mg H_2_O m^-2^ s^-1^ kPa^-1^. These slopes were similar to the slope 1 of the genotypes with BP which were in the range of 23.1–39.8 mg H_2_O m^-2^ s^-1^ kPa^-1^ (Table 2).

### Gas Exchange, Water Potential and Leaf to Air Temperature Differences

**FIGURE 2:**
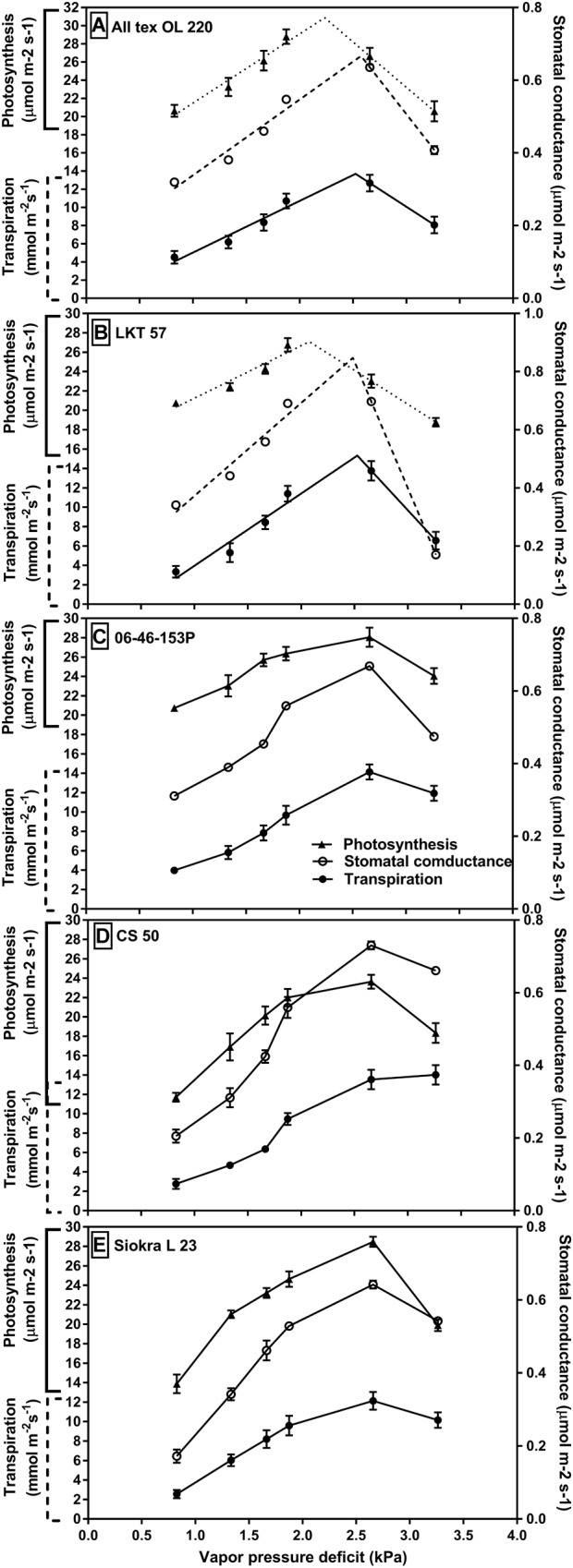
Gas exchange properties of the cotton genotypes with differences in their TR response to VPD. Stomatal conductance (μmol m^-2^ s^-1^), transpiration (mmol m^-2^ s^-1^) and photosynthesis (μmol m^-2^ s^-1^) of the genotypes with limited TR to high VPD **(A)** OL220 and **(B)** LKT 57. These two genotypes were well represented by two segmental linear regression to increase in VPD for their stomatal conductance, transpiration, and photosynthesis with BP at around 2.7 kPa. The genotypes in panels **(C)** 06-46-153 P, **(D)** CS 50, and **(E)** Siokra L23 showed the linear response for their gs, E, and A to the increase in VPD.

Gas exchange, water potential, and leaf temperature were measured in the experiment 3 which was conducted with five selected genotypes from experiments 1 and 2 with and without limited TR. In the third experiment with VPD held between 0.9 and 3.3 kPa for 10 days, gas exchange responses to increased VPD showed differences among the genotypes with and without TR limitation at high VPD (Figure 2). There were slight differences between the VPD maintained in the growth chamber and the VPD obtained in the Li-Cor while taking the measurements. The obtained VPD in Li-Cor for among replicates in each growth chamber also slightly varied. The VPD maintained in each chamber and the range of VPD obtained with Li-Cor for three replicates in that chamber were listed in the Supplementary Table S1. The genotypes ‘OL220’ and ‘LKT 57,’ both with limited TR at high VPD, displayed a limitation in stomatal conductance (*g*_s_,), transpiration (E) and photosynthesis (A) after about 2.2–2.5 kPa (Figures 2A,B). This coincides with the whole TR response measured at different VPDs (0.74–3.02 kPa). The other three genotypes 06-46-153P, CS 50 and Siokra L23 with no limitation in its TR at high VPD also reduced their *g*_s_, E and A at high VPD of 3.3 kPa (Figures 2C–E). Nevertheless, the reduction in OL220 and LKT 57 appeared to be early than other three genotypes (Figure 2). The relation between stomatal conductance and internal CO_2_ was found significantly negative (*P* < 0.03) under high VPD conditions (VPD 2.65 and 3.26 kPa) (Supplementary Figure S1).

**FIGURE 3:**
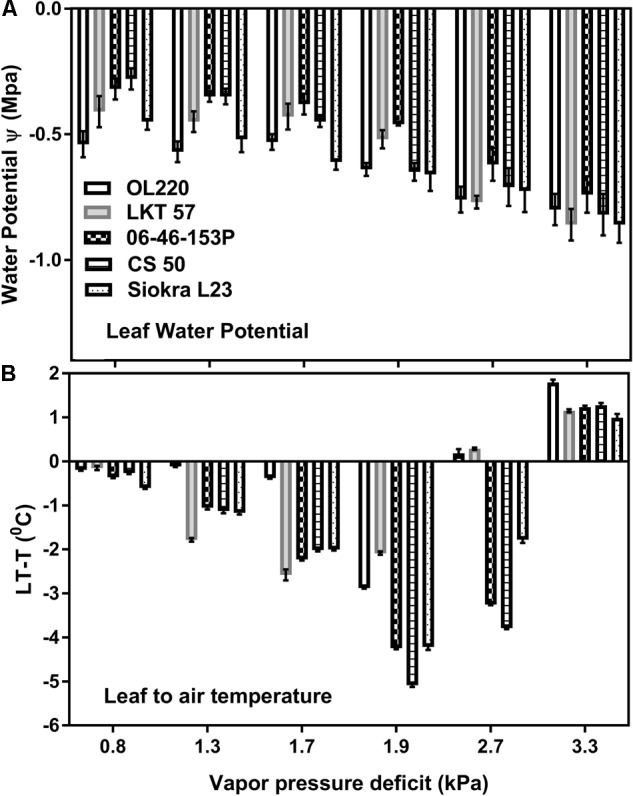
Water potential (ψ) and leaf to air temperature differences (LT-T°C) of the five cotton genotypes to different VPD levels. **(A)** Water potential (Mpa) and **(B)** leaf to air temperature differences (°C) of the genotypes with limited TR and without limited TR to different VPD levels. The first two bars without pattern represent the genotypes OL220 and LKT 57 with limited TR. The other three bars with pattern represents the genotypes without limited TR. The genotypes were significantly different for water potential values (less than *P* < 0.05) at all VPD levels except at 3.3 kPa. The genotypes were significantly (less than *P* < 0.01) different for the leaf to air temperature differences at all VPD levels.

While there were slight differences (*P* < 0.05) among genotypes in the leaf water potential (Ψ) values measured, no conspicuous differences in Ψ among different VPD treatments were noticeable until 3.3 kPa (Figure 3A). The only significant differences were between the low VPD treatment 0.9 kPa and high VPD treatment 3.3 kPa (*P* < 0.01). All genotypes lowered their water potentials at high VPD (3.3 kPa). The leaf to air temperature differences appeared to be high with an increase in VPD and was less at high VPD (3.3 kPa) (Figure 3B). Low leaf temperatures than air temperatures were observed until 1.9 kPa in all genotypes. Apparent high leaf temperature than air temperature was found at high VPDs of 2.7 and 3.3 kPa in limited TR genotypes and at 3.3 kPa in genotypes without TR limitation.

### Stomatal and Epidermal Cell Frequency and Distribution

Stomatal traits were measured in the five selected genotypes in experiment 3 only in the plants exposed to long term VPDs of 0.9 and 3.3 kPa. The VPD influenced stomatal density (SD) on both adaxial and abaxial surface of the cotton leaf (Table 3). The SD was significantly different among the genotypes in plants acclimated to low VPD (0.90 ± 0.05) with an average stomatal density of 131 ± 10 stomata/mm^2^ on the adaxial surface and 250 ± 14 stomata/mm^2^ on the abaxial surface. Similarly, the SD was also varied across the genotypes in plants exposed to high VPD (3.3 ± 0.04) with an average SD of 227 ± 24 and 330 ± 28 on adaxial and abaxial leaf surfaces, respectively. While evaporative demand increased the number of stomata from low to high VPD on the adaxial surface in all genotypes except CS 50, the percentage of increase was found to be low in genotypes with limited TR (71 and 78%) compared to the genotypes with linear TR response (100 and 113) (Table 3). The percentage of increase in SD on abaxial was low in all genotypes compared to adaxial leaf surface, with no significant differences among genotypes. Even though there was a substantial increase in SD on adaxial surface, the genotypes, especially genotypes with limited TR response, reduced their stomatal area (-32.0 to -33.3%) more than the genotypes with linear TR response (-16.9 to -21.6%). All genotypes reduced their stomatal area with an increase in VPD. In contrast, all genotypes increased stomatal area with the increase in VPD on the abaxial surface with a low percentage of increase in genotypes with limited TR (Table 3).

**Table 3:** Adaxial and abaxial leaf surface stomatal density (mm^-2^), stomatal area (μm^2^), epidermal cell density (mm^-2^), epidermal cell area (μm^2^), stomatal aperture length (μm), and stomatal aperture width (μm) of the five cotton genotypes acclimated to low (0.9 kPa) and high (3.3 kPa) VPD environments.

	Stomatal density (mm^-2^)		Stomatal area (μm^2^)		Epidermal cell density (mm^-2^)		Epidermal cell area (μm^2^)		Aperture length (μm)		Aperture width (μm)	
**Genotype**	**Low VPD**	**High VPD**	**Low VPD**	**High VPD**	**Low VPD**	**High VPD**	**Low VPD**	**High VPD**	**Low VPD**	**High VPD**	**Low VPD**	**High VPD**

	**Adaxial leaf surface**											

**OL220**	150 ± 6.02	257 ± 9.32	1025 ± 65.3	725 ± 50.9	516 ± 30.0	351 ± 32.3	1084 ± 110.2	1358 ± 135.0	13.2 ± 1.25	7.1 ± 1.98	3.93 ± 0.08	4.69 ± 0.11
**LKT 57**	160 ± 8.08	285 ± 11.1	850 ± 50.3	700 ± 57.3	496 ± 52.1	331 ± 41.0	1005 ± 100.1	1518 ± 115.2	15.9 ± 1.58	8.18 ± 2.15	3.33 ± 0.05	3.95 ± 0.10
**06-46-153P**	120 ± 8.05	240 ± 10.3	900 ± 52.3	980 ± 40.2	485 ± 63.1	399 ± 56.2	1252 ± 125.3	707 ± 70.5	20.5 ± 2.01	12.7 ± 1.74	3.72 ± 0.09	4.82 ± 0.07
**CS 50**	129 ± 6.52	140 ± 10.2	780 ± 62.3	897 ± 41.8	565 ± 65.2	456 ± 57.3	1166 ± 102.3	771 ± 100.3	16.2 ± 1.02	13.3 ± 1.38	3.87 ± 0.10	4.97 ± 0.09
**Siokra L23**	100 ± 5.29	213 ± 9.30	833 ± 56.3	890 ± 63.2	671 ± 69.3	558 ± 45.0	1139 ± 100.3	930 ± 92.8	19.8 ± 2.05	15.1 ± 1.98	3.77 ± 0.09	4.86 ± 0.10

	**Abaxial leaf surface**											

**OL220**	280 ± 8.02	383 ± 11.1	1050 ± 52.3	1080 ± 40.2	382.6 ± 52.0	410 ± 45.3	727 ± 152.0	1196 ± 112.1	7.09 ± 0.89	9.00 ± 0.99	4.56 ± 0.15	3.01 ± 0.08
**LKT 57**	280 ± 5.29	400 ± 9.30	840 ± 65.3	1100 ± 50.9	472 ± 47.0	518 ± 55.8	822 ± 100.4	1032 ± 112.0	12.1 ± 1.45	14.3 ± 1.56	3.43 ± 0.09	2.89 ± 0.21
**06-46-153P**	244 ± 6.02	343 ± 9.32	933 ± 62.3	1050 ± 41.8	633 ± 63.1	773 ± 67.0	845 ± 92.3	675 ± 62.3	14.1 ± 0.85	15.0 ± 1.46	4.32 ± 0.12	2.41 ± 0.09
**CS 50**	250 ± 8.02	260 ± 10.3	833 ± 50.3	1200 ± 57.3	169 ± 30.3	249 ± 40.2	750 ± 59.3	589 ± 72.4	14.6 ± 0.98	15.7 ± 1.78	3.68 ± 0.10	2.03 ± 0.09
**Siokra L23**	200 ± 6.02	267 ± 8.30	875 ± 56.3	886 ± 63.2	429 ± 42.0	600 ± 56.3	1226 ± 150.1	1086 ± 108.4	13.5 ± 1.05	14.3 ± 2.19	4.80 ± 0.09	4.66 ± 0.11

*The genotypes OL220 and LKT 57 are with limited TR and the other three genotypes (06-46-153P, CS 50 and Siokra L23) are without limited TR at high VPD. The genotypes are significantly different for all the traits at both low and high VPD (P < 0.03 to P < 0.01).*

**FIGURE 4:**
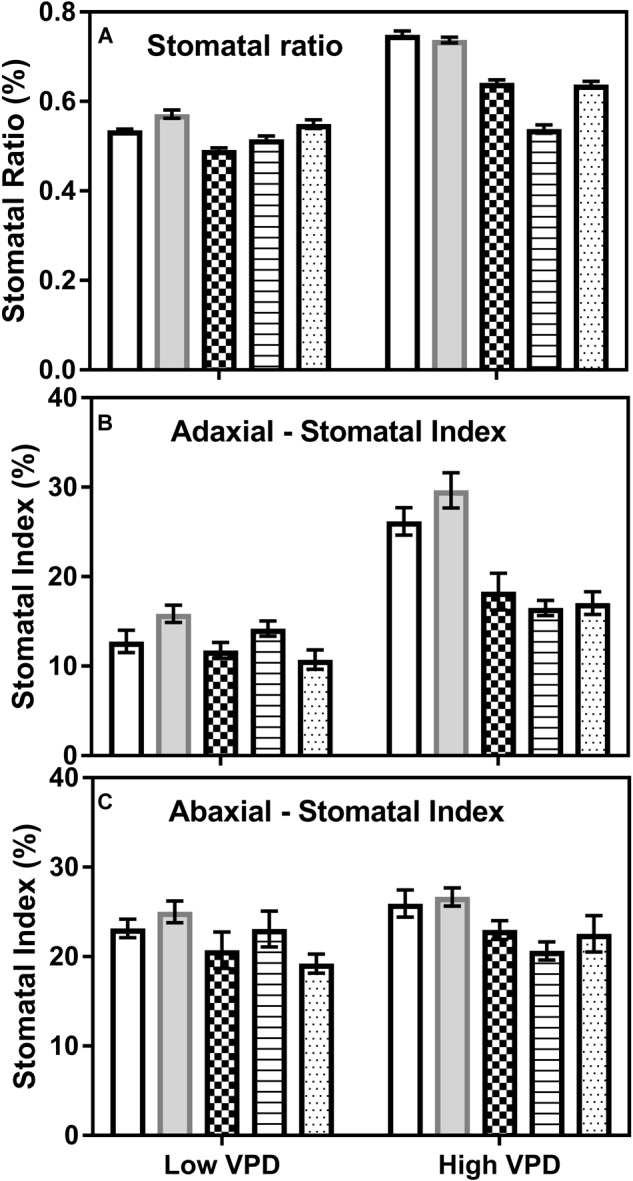
Stomatal ratio and stomatal index of the cotton genotypes with differences in their TR response to VPD on adaxial and abaxial leaf surfaces. **(A)** Stomatal ratio (%), **(B)** adaxial stomatal index (%), and **(C)** abaxial stomatal index (%) of the cotton genotypes grown under low (0.9 kPa) and high (3.3 kPa) VPD environmental conditions. The first two bars in all three panels represent the genotypes with limited TR and other three bars are the data of the genotypes without limited TR. All genotypes were significantly different from each for all parameters under low VPD at *P* < 0.05 and in high VPD at *P* < 0.01.

All cultivars also increased their ratio of stomata from adaxial to abaxial surfaces from low to high VPD with a greater stomatal ratio in OL220 and LKT 57 than the other three genotypes whose TR response to VPD was linear (Figure 4A). The results also showed that the effect on the stomatal index (SI) was different on the adaxial and abaxial surfaces, when VPD significantly increased the SI by 13–51% on the adaxial surface (*P* < 0.001) but marginal or no increase on the abaxial surface (Figures 4B,C). Similarly, VPD also effected stomatal aperture length and width differently (Table 2). High VPD significantly reduced the aperture length in the genotypes OL220 and LKT 57 by approximately 45% on the adaxial surface. This was less than the genotypes with a linear TR response to increasing VPD (Table 3). The response was opposite on the abaxial surface with a slight increase in aperture length in high VPD plants than low VPD acclimated plants (Table 3). However, the elevated VPD had little effect on the aperture width with contrast responses on adaxial and abaxial surfaces. There was a slight increase in width on adaxial surface and reduction on abaxial surface in all genotypes with significantly less (*P* < 0.02) percentage of increase in genotypes with limited TR response on adaxial surface (Tables 3, 4).

**Table 4:** Percentage (%) of increase or decrease in stomatal traits (stomatal density, stomatal area, epidermal cell density, epidermal cell area, aperture length, and aperture width) from low (0.9 kPa) to high (3.3 kPa) VPD conditions of all the five cotton genotypes acclimated to low and high VPD environments.

Genotype	Stomatal density (%)	Stomatal area (%)	Epidermal cell density (%)	Epidermal cell area (%)	Aperture length (%)	Aperture width (%)
	**Adaxial leaf surface**					

**OL220**	71.3 b	–32.0 b	–29.2 c	25.3 b	–46.1 d	19.3 b
**LKT 57**	78.1 b	–33.3 b	–17.6 d	51.1 a	–48.8 d	19.6 b
**06-46-153P**	100 a	–21.6 a	8.88 b	–43.5 e	–38.1 c	29.5 a
**CS 50**	8.52 c	–19.3 a	15.1 a	–33.9 d	–17.7 a	28.4 a
**Siokra L23**	113 a	–16.9 a	6.84 b	–18.4 c	–23.5 b	28.9 a
**LSD**	**23.5**	**3.63**	**3.86**	**4.04**	**4.04**	**3.84**
**P**	**0.004**	**0.001**	**0.001**	**0.001**	**0.0001**	**0.001**

	**Abaxial leaf surface**					

**OL220**	36.7 ab	7.15 d	2.85 d	64.4 a	26.9 a	–33.9 c
**LKT 57**	42.8 a	9.69 d	30.9 b	25.5 b	17.7 b	–15.7 b
**06-46-153P**	40.5 ab	22.1 c	12.5 c	–20.1 d	6.89 c	–44.2 d
**CS 50**	4.23 c	47.5 a	44.1 a	–21.4 d	7.45 c	–44.8 d
**Siokra L23**	33.5 b	39.9 b	1.25 d	–11.4 c	7.18 c	–2.91 a
**LSD**	**4.59**	**3.63**	**2.77**	**3.48**	**3.63**	**3.35**
**P**	**0.001**	**0.001**	**0.001**	**0.001**	**0.001**	**0.001**

*Values denited with similar alphabets are not significantly different based on Tukeys Kramer test. LSD (Least significant difference) and *P* values were calculated for each parameter based on Tukeys Kramer test.*

In addition to stomatal properties, VPD also modified epidermal cell density (ED) and area. Some clear differences (*P* < 0.01) were observed on the adaxial surface in epidermal cell count from low VPD to high VPD and also among the genotypes with differences in their TR response to VPD. The genotypes OL220 and LKT 57 with limited TR to high VPD reduced their ED with -29 and -17%, respectively, when grown under high VPD conditions (Tables 3, 4). All other three genotypes increased their ED marginally (Tables 3, 4). There were no noticeable differences across low and high VPD treatments for their ED on abaxial surface in genotypes OL220 and Siokra L23. The other three genotypes improved their ED with increased VPD (Table 3). The response of epidermal cell area (EA) on adaxial leaf surface to VPD differences was contrast to the course of ED with increased EA in genotypes OL220 and LKT 57 (Table 3). The other three genotypes reduced their EA on both leaf surfaces (Tables 3, 4).

### Correlation of Gas Exchange With Stomatal Properties

A correlation of stomatal ratio with stomatal conductance and transpiration was not observed in plants exposed to high and low VPD. Leaf stomatal index of both adaxial and abaxial surface was not correlated with stomatal conductance and hence with transpiration in plants grown in low VPD environment (Supplementary Figures S2A–D). Nevertheless, there was a significant negative correlation of leaf stomatal index of adaxial and abaxial leaf surface with stomatal conductance and transpiration (P < 0.01–0.03) (Supplementary Figures S2A–D) in high VPD treatment. However, the relationship between stomatal index and photosynthesis was scattered with no significant association.

## Discussion

This study displayed variation among 17 cotton genotypes tested for their transpiration response to the increasing VPD. Even though most of the studied genotypes had consistently increasing TR with increasing VPD, nearly half showed a distinct response by limiting their TR when VPD reached about 2 kPa. The BP, point where TR was limited observed in eight of the cotton genotypes studied is comparable to the VPD for the BP of genotypes in other species in which a limitation in TR to high VPD has been reported (Fletcher et al., 2007; Devi et al., 2010; Gholipoor et al., 2010; Kholová et al., 2010; Schoppach and Sadok, 2013).

The maximum stomatal conductance can be obtained under low evaporative demand conditions (Drake et al., 2013; Dow et al., 2014) which were observed in the present investigation. The genotypes OL220 and LKT 57 with limited TR at high VPD, associated with reduced stomatal conductance also constrained their photosynthetic rate (Figure 2). Often, this reduction in transpiration and photosynthesis may be counterbalanced by conserving the water with limited TR to high VPD for use later in the growing season (Sinclair et al., 2017). However, in this study, the genotypes with linear VPD- TR response 06-46-153P, CS 50 and Siokra L23 also reduced their stomatal conductance and photosynthesis at VPD of 3.3 kPa. Relatively, the reduction in genotypes with limited TR was at early VPD (2.7) than genotypes without TR limitation (3.3 kPa). The VPD in growing season on most of the days may possibly go beyond 2 kPa. At this time, the major consequence of decreasing stomatal conductance and subsequently the transpiration in the midday under high VPD is conservation of soil water. This would effectively maximize the transpiration efficiency by minimizing the percentage of the transpiration that usually arises during periods of high VPD (Sinclair et al., 2005). Reduction in photosynthesis followed abruptly by the decrease in stomatal conductance because of the fundamental link between water vapor and CO_2_ exchange in leaves (Tanner and Sinclair, 1983). Nevertheless, if there should be any incidence of late season drought, the genotypes with limited TR have the possibility of utilizing the water conserved by limiting the TR which would assist in improving yield than the genotypes without limited transpiration. In fact, in simulation studies of sorghum production in Australia and soybean in the United States showed a yield increase of about 75% of the seasons as a result of the limited transpiration at high VPD (Sinclair et al., 2005, 2010). However, the trait resulted in yield loss under wet conditions confirming the benefit of the trait under mild and severe drought conditions (Sinclair et al., 2010). Future simulation/crop modeling studies should be conducted in cotton to assess the utility of this trait and yield improvement in environments where mild or severe stress occurs.

Regulation of stomata to VPD is a process, by which plants adjusts their transpiration during daytime. Despite several studies, the mechanism for the stomatal response to VPD are poorly understood (McAdam and Brodribb, 2015). Some studies have suggested limited TR due to hydraulic limitation and the involvement of water channel proteins (Sinclair et al., 2017). Stomatal regulation of leaf water balance has been proposed to be controlled by active metabolic processes along with passive hydraulic process (McAdam and Brodribb, 2014). Recent studies had observed the expression of ABA synthesizing genes in guard cells and regulating stomatal responses to VPD (Waadt et al., 2014; McAdam and Brodribb, 2015). Apparently, these metabolic processes are prompted by low leaf water potential due to ABA (McAdam et al., 2016) and might result in a limitation in the TR. There were only marginal differences in leaf water potential with an increase in VPD until 3.3 kPa. The insensitivity to any decreases in leaf water potential is consistent with the observations in pea where leaves had to be exposed to more than -1 Mpa of pressure to induce a sufficient loss in turgor (McAdam and Brodribb, 2014). In this investigation, all genotypes lowered their Ψ at 3.3 kPa to the levels that were non-damaging. This is possibly due to feed forward response of the stomata to high evaporative demand (Bunce, 1997; Buckley, 2005). Reduction in stomatal conductance to high VPD caused in maintaining leaf Ψ to lower the damage to leaf.

A change in relative humidity and the concomitant change in TR often results in leaf temperature changes (Talbott et al., 2003). As VPD increased until 2.7 in limited TR genotypes and 3.3 in other genotypes, leaf temperature to air temperature (LT-T) ranged from a fraction of 1 to negative, probably because of increased transpiration cooling. However, at high VPD the difference between leaf to air temperature was positive, presumably due to the reduction in stomatal conductance and transpiration. The decrease in TR might have resulted in an increase of leaf temperature that caused the positive leaf to air differences (Pallas et al., 1967). An increase in temperature will have an impact on photorespiration causing temporary build up in internal CO_2_ Ci. Increased stomatal sensitivity to Ci was considered as a primary cause of reduced stomatal conductance under high VPD in wet soils (Bunce, 1997, 2003, 2007). The adjustments in the stomatal conductance are mainly to keep Ci/Ca at nearly a constant ratio, which results in a steady or reduced TR (Bunce, 1997, 2003, 2007), based on the adjustments. In the current study under high VPD conditions, where the TR was limited, a negative relation was observed between Ci and stomatal conductance (Supplementary Figure S1). As the data points in the study are small, the theory requires further examination including many data points.

The possibility of the contribution of stomatal traits in the expression of limited transpiration to high VPD was studied. Earlier studies have suggested the modification of stomatal properties by improving the density and shrinking the cell size in response to water deficit as an adaptation strategy (Martínez et al., 2007). In previous studies, in addition to other environmental factors, the effect of VPD on stomatal density and area was also investigated (Luomala et al., 2005; Carins Murphy et al., 2014). An increase in stomatal density and decrease in stomatal area from low to high VPD was observed especially on adaxial leaf surface in genotypes with limited transpiration response (Table 2). While stomatal density drives the partitioning of conductance among leaf sides, in general, stomatal size links to the *g*_s_ with larger pores promoting *g*_s_ (Fanourakis et al., 2015). Therefore, the decrease in stomatal size with the effect of high VPD corresponds to the decrease in *g*_s_ and subsequently reduced TR in the current study.

Plants in response to environmental stress not only alter stomatal frequency (Xu and Zhou, 2008), but also change stomatal aperture size anatomically (Casson and Hetherington, 2010). All genotypes reduced their adaxial and abaxial stomatal length to high VPD but increased their width. The reduction in the stomatal aperture length to control transpiration at elevated temperature in maize leaf and different environmental variables in Arabidopsis was reported (Lomax et al., 2009; Zheng et al., 2013). Nevertheless, all genotyped reduced their stomatal aperture length to increase in VPD, with the high percentage of reduction in limited TR genotypes. Additionally, high VPD also modified stomatal ratio by increasing the ratio with no observed relation with any of the gas exchange parameters measured.

The previous study in maize had reported that the elevated VPD enhanced epidermal cell expansion in leaves but had little effect on the epidermal cell division (Tardieu et al., 2000). This suggests that the decrease of epidermal cell density in the high VPD than the low VPD treatment especially in TR limiting genotypes in the current study was mainly due to the greater expansion of the individual epidermal cells, rather than greater differentiation to stomatal cells under high VPD treatment. Our results also confirmed that the average size of the individual epidermal cells was significantly larger in the high VPD treatment than in the low VPD (Table 2) on the adaxial leaf surface. Correspondingly, some studies have identified that the plants adapt to various environmental variables by regulating their stomatal densities through modifying their epidermal cell density rather than stomatal numbers (Driscoll et al., 2006). The alteration of the stomatal index to high VPD also well related with the transpiration and stomatal conductance (Supplementary Figure S2).

## Conclusion

The results of the present investigation indicated a range in the response of TR to VPD in cotton genotypes. Several of the genotypes exhibited a fairly limited TR at high VPD and while some demonstrated increasing TR. The limitation in TR at high VPD would contribute to water saving in the soil profile when water is available. This conserved water would be useful to the plants at the time of maturity and therefore for the yield improvement when late season drought develops. The results here can also be useful in the breeding program for the development of genotypes with optimized limited TR to utilize in water-limited environments. Field validation is required before these breeding strategies are applied to the genotypes limiting TR studied here. Future work needs the confirmation of the limited TR of the tested cotton genotypes in the current study in the field in a wide range of VPD. The genotypes also need to be tested for water conservation trait for the period of flowering through maturity.

The study here also indicates that it may be possible to adapt the transpiration response at elevated VPD by modifying their stomatal properties. The genotypes with limited exposed to the range of VPD (0.9–3.3 kPa) for longer time also retained their limited TR even after acclimatization. The genotypes with limited TR acclimated to low and high VPD reformed their stomatal traits differently. Stomatal and epidermal cells have a higher plasticity in response to a larger range of VPD, and these parameters are clearly associated with differences in transpiration response to high VPD in cotton genotypes resulting limited transpiration. The substantial changes in stomatal development might have facilitated to maintain transpiration homeostasis in cotton genotypes grown under contrasting VPD environments.

## Author Contributions

MD and VR conceived and designed the experiments. MD conducted the experiments, collected and analyzed the data. MD and VR wrote the manuscript.

## Conflict of Interest Statement

The authors declare that the research was conducted in the absence of any commercial or financial relationships that could be construed as a potential conflict of interest.
