# Supplementary material for: Transpiration Response of Cotton to Vapor Pressure Deficit and Its Relationship With Stomatal Traits
Source: Front Plant Sci. 2018 Oct 30;9:1572. doi: 10.3389/fpls.2018.01572 (PMC6218332; doi:10.3389/fpls.2018.01572)
Supplement: Supplementary file 1 [file Data_Sheet_1.pdf]

Supplementary table S1

VPD in the growth chamber (kPa)	Range of VPD in Li-cor (kPa)	Average VPD in Li-cor (kPa)
0.83±0.01	0.78 to 0.82	0.80±0.014
1.33±0.01	1.22 to 1.42	1.34±0.074
1.66±0.02	1.53 to 1.66	1.60±0.044
1.87±0.01	1.87 to 1.91	1.89±0.016
2.65±0.01	2.54 to 2.69	2.63±0.056
3.26±0.02	3.30 to 3.42	3.38±0.023

Supplementary table S1: Vapor pressure deficit (VPD)±S.E. maintained in the growth chamber experiment 1. Range of VPD obtained in Li-cor during gas exchange measurements for the three replicates in each chamber and the average VPD±S.E..
