# Supplementary material for: Transpiration Response of Cotton to Vapor Pressure Deficit and Its Relationship With Stomatal Traits
Source: Front Plant Sci. 2018 Oct 30;9:1572. doi: 10.3389/fpls.2018.01572 (PMC6218332; doi:10.3389/fpls.2018.01572)
Supplement: Supplementary file 2 [file Data_Sheet_2.pdf]

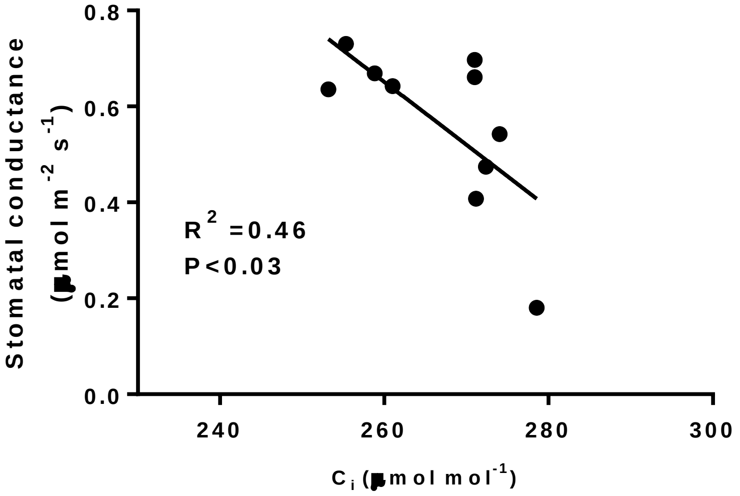

Supplementary Figure S1: Relationship between Internal carbon (Ci) and stomatal conductance in five cotton genotypes in experiment 3 under 2.65 and 3.26 VPD conditions.
