# Supplementary material for: Transpiration Response of Cotton to Vapor Pressure Deficit and Its Relationship With Stomatal Traits
Source: Front Plant Sci. 2018 Oct 30;9:1572. doi: 10.3389/fpls.2018.01572 (PMC6218332; doi:10.3389/fpls.2018.01572)
Supplement: Supplementary file 3 [file Data_Sheet_3.pdf]

Supplementary figure S2

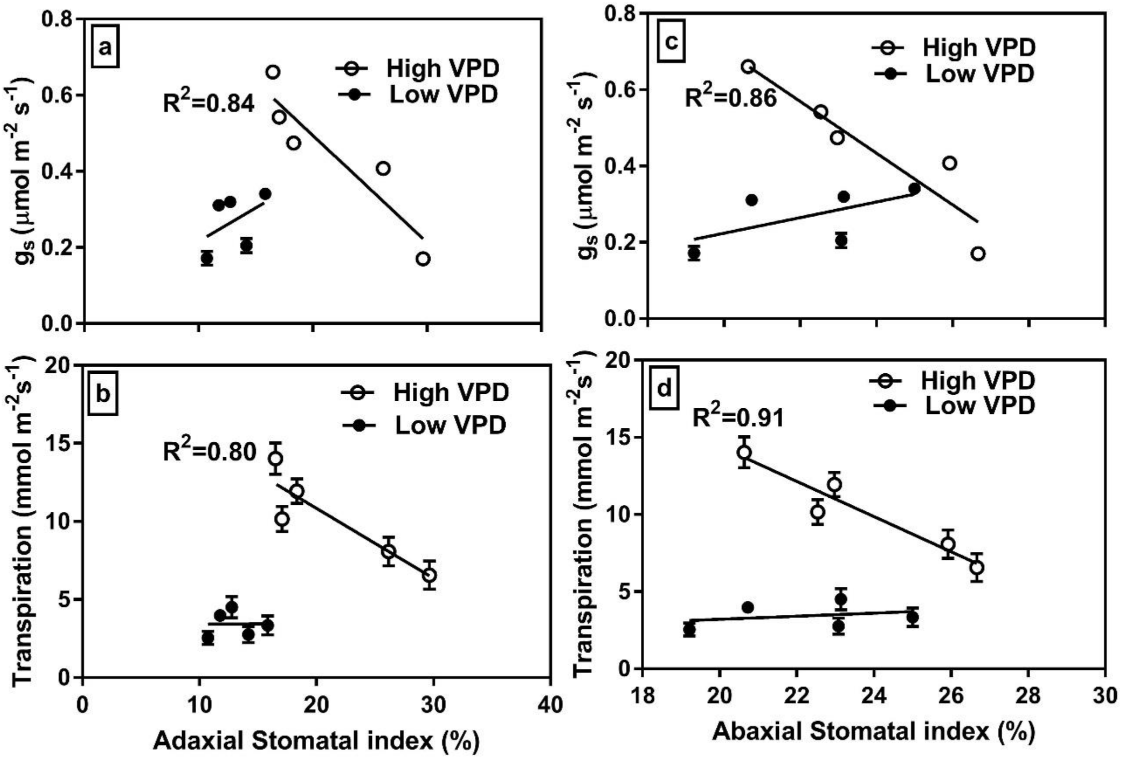

Supplementary figure S2: Relationship of Adaxial and Abaxial stomatal index with stomatal conductance (gs) and transpiration in five cotton genotypes

a) Stomatal conductance gs (μmolm^{-2}s^{-1}) and b) Transpiration (mmolm^{-2}s^{-1}) panels represents relationship with adaxial stomatal index and c) gs (μmolm^{-2}s^{-1}) and d) Transpiration (mmolm^{-2}s^{-1}) with abaxial stomatal index in five cotton genotypes. The closed circles are the data obtained under low VPD and open circles under high VPD. In all panels the correlation was significant only for high VPD conditions (R^2 from 0.80 to 0.91, $P<0.01$).
